# Genetically modified chickens as bioreactors for protein-based drugs

**DOI:** 10.3389/fgeed.2024.1522837

**Published:** 2025-01-07

**Authors:** Shujuan Meng, Aijun Miao, Sen Wu, Xuguang Du, Fei Gao

**Affiliations:** ^1^ Frontiers Science Center for Molecular Design Breeding (MOE), State Key Laboratory of Animal Biotech Breeding, College of Biological Sciences, China Agricultural University, Beijing, China; ^2^ Sanya Institute of China Agricultural University, Sanya, China

**Keywords:** protein expression, bioreactor, glycosylation, CRISPR/Cas9, gene editing

## Abstract

Protein drug production encompasses various methods, among which animal bioreactors are emerging as a transgenic system. Animal bioreactors have the potential to reduce production costs and increase efficiency, thereby producing recombinant proteins that are crucial for therapeutic applications. Various species, including goats, cattle, rabbits, and poultry, have been genetically engineered to serve as bioreactors. This review delves into the analysis and comparison of different expression systems for protein drug production, highlighting the advantages and limitations of microbial, yeast, plant cell, and mammalian cell expression systems. Additionally, the emerging significance of genetically modified chickens as a potential bioreactor system for producing protein-based drugs is highlighted. The avian bioreactor enables the expression of target genes in ovarian cells, resulting in the production of corresponding gene expression products in egg whites. This production method boasts advantages such as a short cycle, high production efficiency, low research costs, and the expression products being closer to their natural state and easier to purify. It demonstrates immense potential in production applications, scientific research, and sustainable development. The utilization of advanced gene editing technologies, such as CRISPR/Cas9, has revolutionized the precision and efficiency of generating genetically modified chickens. This has paved the way for enhanced production of recombinant therapeutic proteins with desired glycosylation patterns and reduced immunogenic responses.

## 1 Introduction

The swift evolution of biotechnology has facilitated the development of an expanding array of proteins and peptides as therapeutic agents for diverse pathological conditions. Proteins, as intrinsic biomacromolecules, exhibit considerable clinical promise in treating a multitude of diseases, encompassing cancer, immune disorders, and metabolic abnormalities. These protein-based pharmaceuticals can be categorized into peptides, monoclonal antibodies, genetically engineered antibodies, and recombinant vaccines (Younis Nadeem et al., 2022; Behrendt and Wedemeyer, 2022). In contrast to small-molecule drugs, therapeutic proteins boast advantages such as ease of programming, functional diversity, high specificity, minimal toxicity, and exceptional efficacy (Liu et al., 2024). Notably, numerous protein drugs currently available in the market have demonstrated remarkable therapeutic outcomes in managing AIDS, cancer, hepatitis, diabetes, chronic pain, and other conditions. Over the past decade, the U.S. Food and Drug Administration (FDA) has approved over 100 commercial drugs, many of which are protein-based. Consequently, the pharmaceutical industry has witnessed a substantial surge in the demand for protein production.

Recombinant protein drugs are biologically active protein products that are manufactured utilizing recombinant DNA technology. This process involves optimizing and modifying the gene encoding the target protein. It then involves introducing the target gene into appropriate host cells using a specific vector for expression, and subsequently extracting and purifying the target protein. These drugs are employed to address the deficiency of functional proteins in the body resulting from congenital genetic defects or acquired diseases. Recombinant protein drugs encompass polypeptide hormones, cytokines, plasma protein factors, recombinant enzymes, and fusion proteins. The heightened demand for protein drugs in the treatment of cancer, metabolic disorders, and other diseases, as well as their use in biological research, has led to an increasing focus on finding suitable hosts for the large-scale production of highly active recombinant medicinal proteins with shorter generation times (Joseph et al., 2016; Evan and Robinson, 2017).

With the progression and refinement of recombinant protein production technology, various innovative recombinant protein drugs are continually being developed. In the early 1980s, human insulin was successfully produced using recombinant bacteria and remains a staple in diabetes management today, with a substantial market presence. Currently, most recombinant protein drugs are produced through bacterial cell culture systems (Overton, 2014). Despite ongoing challenges in the large-scale development of recombinant proteins and peptides as therapeutic agents, researchers worldwide have relentlessly pursued exploration in this field since the advent of insulin production. In recent decades, the manufacturing of recombinant therapeutic proteins and monoclonal antibodies has revolutionized the pharmaceutical landscape. Mammalian cell culture systems have emerged as the primary platform approved for the production of human recombinant protein drugs, albeit with the constraint of high cultivation costs (Sanchez-Garcia et al., 2016). In comparison, the production of transgenic animals promises greater cost-effectiveness, particularly given the significant advancements achieved in the targeted expression of therapeutic proteins in the mammary glands of dairy cows, sheep, and goats (Rudolph, 1999; Houdebine, 2000; Keefer, 2004; Wilfried and Niemann, 2004). In 2009, the FDA approved the first pharmaceutical protein derived from a transgenic animal. Subsequently, a biotherapeutics company developed transgenic goats that produce antithrombin in their milk using a mammary-specific promoter (Kling, 2009). Genetically modified chickens also hold significant potential in recombinant therapeutic production due to their short breeding cycles, straightforward management, and high productivity (Ken-ichi and Iijima, 2013). In 2015, the FDA approved a novel drug named “Kanuma.” This drug is a recombinant version of human lysosomal acid lipase (rhLAL), designed to replenish the deficient enzyme in patients and thereby restore normal metabolism. This successful case underscores the immense potential of genetically modified chickens in the field of biopharmaceutical production (Sheridan, 2016). Furthermore, the enhancement of various legislative frameworks will contribute to the advancement of transgenic animal protein production.

This review aims to analyze and compare different production methods of recombinant pharmaceutical proteins, with a detailed elaboration on the principles, advantages and development status of avian oviduct as a bioreactor. Furthermore, it seeks to offer valuable guidance for the cost-effective and efficient production of precious and urgently needed pharmaceutical proteins.

## 2 Methods for producing recombinant protein drugs

In the realm of recombinant protein drug production systems, prokaryotic and eukaryotic cell culture systems have historically dominated, with *Escherichia coli* (*E. coli*), yeast, and Chinese hamster ovary (CHO) cell lines serving as the primary expression systems. Recombinant therapeutic proteins expressed through these systems have successfully been commercialized worldwide. In Table 1, we compared the production efficiency and cost-effectiveness of various bioreactor systems. Detailed descriptions of the specific production methods, along with their advantages and disadvantages, were provided in separate chapters.

**TABLE 1:** Comparison of production efficiency and cost-effectiveness among different bioreactor systems.

Bioreactor type	Efficiency^a^	Cost-effectiveness^b^	Production features
Microbial reactors	medium	Moderate to high	Suitable for producing eukaryotic proteins with limited post-translational processing
Plant bioreactors	Moderate to high (depending on gene expression.)	Low	Low production costs, high safety, relatively low expression levels of recombinant proteins, and downstream processing costs that vary based on protein expression levels
Cell bioreactors	Higher	medium	The production process is relatively streamlined, easy to operate and manage, yet challenging to scale up
Animal bioreactors	High	Low to medium (depending on production costs and downstream processing costs)	Production of pharmaceutical proteins with full biological activity yields high volumes but requires a long waiting period

^a^ The calculation method for production efficiency is output/time.

^b^ The calculation method for cost-effectiveness is cost/output.

### 2.1 Microbial expression system

#### 2.1.1 Bacterial expression system

Currently, various types of recombinant proteins have been studied and marketed using microbial expression systems. Many pharmaceutical proteins are produced in microbial fermenters because they can grow rapidly at any scale. Among them, *E. coli* plays an important role in vaccine development due to its simple cultivation and convenient operation. Moreover, the effective secretion of recombinant proteins in the periplasmic space of *E. coli* can improve the solubility of proteins expressed in *E. coli*. At present, the development of various types of recombinant protein vaccines based on recombinant protein monomers, recombinant protein aggregates, virus-like particles, etc., has been achieved in the *E. coli* expression system. Cambridge Bio has utilized the *E. coli* expression system to produce the Feline Leukaemia Virus (FeLV) gp70 surface glycoprotein, developing the first batch of successfully applied veterinary recombinant protein vaccines (Marciani et al., 1991). Some recombinant proteins are more readily obtained in their active forms from *E. coli* hosts. A notable example is the recombinant hepatitis E vaccine, Hecolin, which is the world’s first hepatitis E vaccine and the first successfully marketed virus-like particle vaccine from an *E. coli* host, filling the gap in the research field of hepatitis E vaccines (Shao et al., 2015). In addition, the human recombinant protein vaccines that have been completed and are being developed in *E. coli* hosts include the bivalent human papillomavirus vaccine Cecolin (Zhao et al., 2022), Recombinant serogroup B meningococcal vaccine (Yue et al., 2022), Influenza A (H1N1) vaccine (Hoang et al., 2022), Malaria vaccine DIAID (Hu et al., 2022). However, it is well-established that bacterial cell culture systems possess poor glycosylation and post-translational modification capabilities (Swartz, 2001; Gomord and Faye, 2004; Houdebine, 2009; Wang Haoyi et al., 2013; Joel et al., 2014; Overton, 2014). Certain proteins, even naturally occurring ones, must undergo post-translational modifications to attain functional structures and maintain stability within the body. These modifications primarily encompass protein folding, cleavage, subunit binding, gamma-carboxylation, and glycosylation. Consequently, bacterial expression systems are constrained by their limited modification abilities, preventing them from synthesizing complex proteins such as monoclonal antibodies or coagulation factors.

#### 2.1.2 Yeast expression system

The development of eukaryotic expression systems has largely broken through the bottleneck of complex protein expression in bacteria. This system has been widely used in structural and biophysical research, functional analysis, biomarkers, and drug production. In addition, recombinant protein drugs produced using eukaryotic expression systems have gradually become commercialized and industrialized (Fatima et al., 2021; Mastrangeli et al., 2021). Studies have shown that transgenic yeast is capable of expressing foreign genes encoding glycosylating enzymes, thereby being able to secrete large amounts of recombinant proteins whose carbohydrate structures are almost similar to those in human proteins (Kumar Das et al., 2024). In these aspects, yeast cells offer certain advantages over bacteria. Additionally, some yeast expression systems possess exocrine signal sequences that enable the secretion of expressed exogenous proteins outside the cell, facilitating easy purification. This suggests that yeast has significant potential as an important system for the production of pharmaceutical proteins. In certain host yeast strains, therapeutic glycoproteins with glycosylation have already been produced (Azimzadeh Irani et al., 2016). However, despite yeast’s capability to perform N-glycosylation and O-glycosylation, it exhibits significant differences in glycosylation patterns compared to human cells. The recombinant proteins secreted by yeast undergo α-1,3-mannosylation, leading to increased protein immunogenicity and shortened half-life. Yeast cells and human cellular systems possess distinct post-translational modifications (Maksimenko et al., 2013). The recombinant hepatitis B vaccine obtained from yeast cells is unable to form the necessary disulfide bonds, and this modification defect makes it not feasible for all proteins. Thus, Consequently, neither bacteria nor yeast can carry out certain post-translational modifications necessary for human therapeutic proteins to fully exert their biological activities (Dana and Krummen, 2002). Yet, many potential therapeutic proteins require specific post-translational glycosylation modifications *in vivo* to become active (Fussenegger et al., 1999). For example, α1-antitrypsin is a soluble factor that is deficient in respiratory diseases such as emphysema and requires glycosylation of natural proteins to play a therapeutic role (Clark, 1998). The shortcomings of these culture systems have prompted researchers to diligently seek potential alternatives to traditional cell production systems.

### 2.2 Plant cell expression system

In addition to bacteria (Huang et al., 2012) and yeast (Nielsen, 2013), It has been reported that other host-fungi (Nevalainen and Peterson, 2014) and plants (Julian et al., 2003) can also produce exogenous recombinant proteins. Significant progress has also been made in the development of genetically modified plants for drug production (Julian et al., 2003). A method that enables the production of recombinant human proteins in the leaves or seeds of these plants. The use of plant cell expression systems for large-scale production of protein-based drugs is a relatively mature biopharmaceutical technology. This method boasts excellent safety and scalability, while also offering a rapid production process and lower costs. Based on plant endogenous specific promoters, exogenous recombinant proteins can be expressed in the leaves, seeds, or both of plants. The advantages of this system are low production costs and the absence of mammalian viral sequences and pathogen contamination. It is worth mentioning that the post-translational modification of proteins in plant cells is basically similar to that in animal cells. It can effectively enable the expressed proteins to fold and bind subunits correctly, just like animal cells, and can also add carbohydrates to the protein chain. In addition, the plant expression system can also mediate the folding and formation of disulfide bonds with the assistance of molecular chaperones, thereby increasing the degree of glycosylation of plant-derived molecules. For instance, fucose and xylose-free glycoproteins have been successfully obtained in whole plants or plant cell lines through the use of RNA interference or genome editing methods (Hanania et al., 2017). A new expression system based on plant cell suspension packages has been developed. After amplification, downstream extraction and purification, it can also be used for the production of protein drugs (Navarre et al., 2017). However, there are some important issues that must be considered and resolved before pharmaceutical proteins produced in plant reactors can enter the market. Firstly, in plants, the sugar groups added to proteins are different from those in animals, and the structures of most proteins are not exactly the same. Yet, the structures of many protein drugs required by humans must adapt to the human environment. Secondly, and more importantly, the use of large-scale plant production as a tool for the production of exogenous proteins must consider the practical issues of environmental safety. This method has the risk of introducing transgenes that spread to wild populations and cause food crop contamination. Additionally, there may be viral invasion during the expression of proteins in transgenic plants, and the public’s acceptance of transgenic plants is also an issue that must be considered.

### 2.3 Mammalian cell expression system

Significant progress has been made in the production of exogenous proteins using various animal cell lines, such as Chinese hamster ovary (CHO) cells (Kim et al., 2012; Jahir and Lewis, 2015), mouse myeloma (NS0) cells (Li et al., 2010), human amniotic cell line (1G3) (Silva et al., 2015) and human embryonic kidney cell (HEK-293) culture system (Lan Pham et al., 2006). Mammalian cell expression systems have emerged as the primary production systems for recombinant protein drugs used in clinical applications. Over half of the commercially available biopharmaceuticals and hundreds of candidates in clinical development are produced using mammalian cell expression systems. Human cell lines have become a new and powerful alternative for the production of recombinant proteins. These cells are able to produce recombinant proteins with post-translational modifications that are closer to natural proteins, produce proteins with human-like glycosylation patterns, and avoid immunogenic responses to non-human epitopes. Mammalian cells, particularly CHO (Chinese Hamster Ovary) cells, have been successfully used as reaction systems for nearly two decades. Human tissue plasminogen activator was the first recombinant therapeutic protein successfully produced in a CHO cell line and approved for clinical use (Wang and Guo, 2020). Subsequently, an increasing number of therapeutic proteins have been produced through mammalian cell cultures. With the optimization of cell culture techniques and continuous improvements in production processes, the technology for producing valuable recombinant proteins in this expression system has rapidly advanced. The main advantage of these mammalian cell culture systems is that these cell lines have appropriate post-translational modifications. These modifications occur to varying degrees, ensuring that the exogenous proteins obtained in this culture system can achieve relatively correct structures. Although the cell culture system has made a great contribution to the production of recombinant proteins, it still has some limitations. Compared with other production systems, mammalian cell expression technology is complex, and the special small molecule components of some culture media are relatively expensive, resulting in higher production costs for culturing cells (Houdebine, 2009; Kwon et al., 2018). Additionally, due to varying proliferation rates, mammalian cells exhibit relatively low production levels and may be susceptible to contamination from viruses originating from the cells themselves or other sources. In practice, there is a high demand for pharmaceutical proteins, yet their production is constrained by expensive cultivation costs and the capacity to produce recombinant proteins on a large scale. Furthermore, despite improvements in animal cell culture conditions, the production of recombinant proteins and their glycosylation processes may still be unstable.

### 2.4 Transgenic animal bioreactor

**FIGURE 1:**
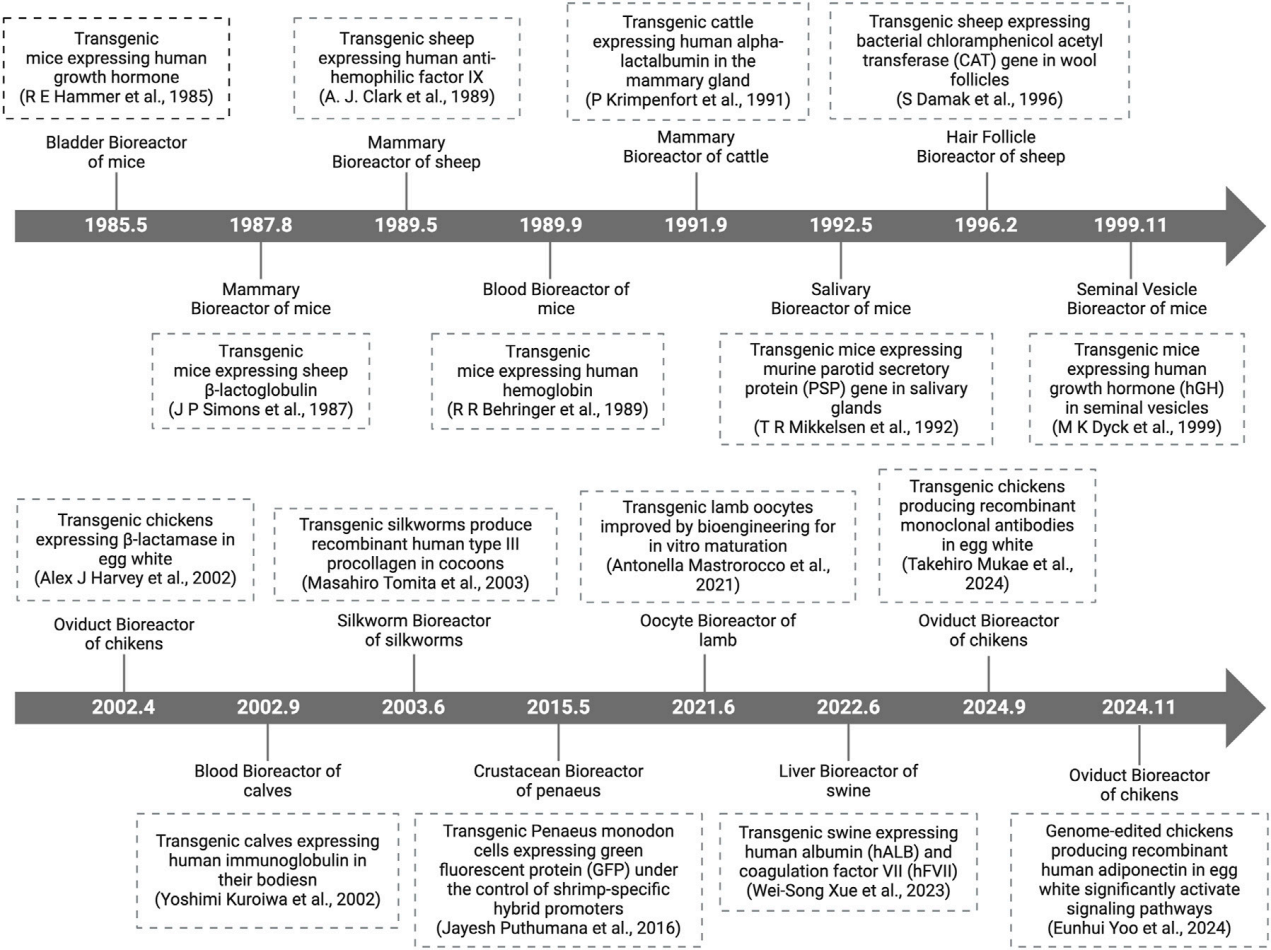
Timeline of key dates for the development of different bioreactor systems. PSP, parotid secretory protein; CAT, chloramphenicol acetyl transferase; hGH, human growth hormone; GFP, green fluorescent protein; hALB, human albumin; hFVII, human coagulation factor VII.

With the discovery and continuous development of transgenic technology, the history of transgenic animal bioreactors for producing pharmaceutical proteins has evolved from initial establishment to widespread application. Many laboratories have successfully cultivated various types of transgenic animals, such as mice, rats, rabbits, sheep, goats, pigs, and cows, and have obtained medically valuable protein drugs from the blood and milk of these animals. In Figure 1, we present the historical milestones of different bioreactor systems. Subsequent content provides a more detailed description of avian bioreactors.

#### 2.4.1 Mammalian bioreactor

Shortly after the first methods for genetically modifying mice were developed, researchers proposed the possibility of using transgenic animals to produce therapeutic proteins. Various transgenic animal species have the ability to produce recombinant proteins in their mammary glands or other secretory organs (Houdebine, 2009). The animal mammary gland bioreactor utilizes transgenic technology to introduce exogenous genes into the animal genome and target their expression specifically to the animal’s mammary gland. By harnessing the natural and efficient protein synthesis and secretion capabilities of the animal’s mammary gland, valuable products, especially humanized proteins, can be produced in the animal’s milk (Zhang et al., 2023). The mammary gland bioreactor is regarded as a technological innovation in the production of pharmaceutical proteins due to its advantages such as efficient expression, low production cost and the ability to synthesize proteins with structures close to natural proteins. It is generally considered as a superior choice and currently the only bioreactor that can be commercialized internationally. The recombinant human antithrombin III (ATryn) produced by GTC in the United States using a goat mammary gland bioreactor became the first bioengineered drug approved for marketing by the European Medicines Agency and the US FDA. Milk is currently the most mature system for producing recombinant proteins from genetically modified organisms (Houdebine, 2000). However, the production of recombinant proteins in milk requires a long and complex process. The growth of dairy cows takes a long time, and the purification of pure target proteins from large amounts of milk protein and fat requires complex downstream processing steps (Ivarie, 2003; Zivko and Woodard, 2004). In theory, similar systems may also exist in blood, milk (Houdebine, 2002), egg white (Zhu et al., 2005; van de Lavoir et al., 2006; Lillico et al., 2007), seminal plasma (Dyck et al., 2003), urine, silk glands (Royer et al., 2005), and insect larval hemolymph (Markaki et al., 2007). However, its application is greatly limited by problems such as random integration of exogenous genes and unstable expression of recombinant proteins. Furthermore, due to the constraints of some zoonoses and other factors, the continuous production of pharmaceutical proteins in mammalian bioreactors requires repeated and complicated quarantine procedures to ensure their biosafety, making production time-consuming and labor-intensive. It is very unfavorable for the production of some precious and urgently needed pharmaceutical proteins. The mammary cell machinery may be saturated and unable to fully glycosylate additional proteins. The recombinant protein ATryn (human antithrombin II) produced in goat milk contains less sialic acid than its natural counterpart (Edmunds et al., 1998). Likewise, human inhibitor C1 produced in rabbit milk was not fully sialylated (Koles et al., 2004). The transfer of glycosylation enzyme-encoding genes has improved the transfer of carbohydrates to proteins synthesized in yeast (Hamilton et al., 2003), as well as enhanced carbohydrate transfer in CHO cells (Weikert et al., 1999).

#### 2.4.2 Avian bioreactor

The technological pathway for producing therapeutic proteins using animal bioreactors had already matured, and the first products have begun to be applied to the market. Despite there are more and more options for the production of therapeutic proteins, it has been recognized that the generation time for large animals is relatively long, and each litter typically consists of a small number of offspring. This poses challenges for the production of urgently needed and valuable pharmaceutical proteins, as the resources required for commercial-scale production constitute a bottleneck. Utilizing the avian oviduct as an expression system to produce exogenous proteins in avian eggs offers significant advantages for the production of specific therapeutic protein drugs. These advantages include lower production costs compared to cell culture or transgenic mammalian expression systems, faster scale-up speeds, and a higher degree of protein modification sophistication (Rachel et al., 2018). For the production of medicinal proteins, post-translational modifications of many proteins are essential for their function. If a protein is not properly modified, it may have a short half-life in the patient, and the therapeutic effect may be poor. Glycosylation plays an crucial role in the final structure, solubility, stability, folding, localization, biological activity and half-life of proteins (Lei et al., 2016). The incorporation of glycoproteins into the final protein structure varies depending on the host (mammalian, insect, bacterial or plant cells) and can directly affect the immunogenicity of the protein (Raju, 2003; Lei et al., 2016). Lack of specific glycosylation modification can lead to immunogenicity of the produced recombinant protein. Therefore, before the pharmaceutical protein enters the real clinical application, structural assessment must be conducted on the target protein to determine whether appropriate glycosylation *in vitro* is necessary to achieve the desired therapeutic effect. Studies have confirmed that human monoclonal antibodies and therapeutic proteins produced in egg white contain almost correct post-translational modifications, making them effective therapeutic agents (Zhu et al., 2005; Mizutani et al., 2012; Kojima et al., 2014). Hence, selecting a suitable host for the production of recombinant proteins is an important criterion for the production of biomaterials. A study on the glycosylation of immunoglobulins across different species has highlighted species-specific differences in the sialylation of N- and O-linked oligosaccharides, which are major forms of post-translational glycosylation (Raju et al., 2000). In humans, the 1,3-galactosyltransferase enzyme is inactive, and B lymphocytes produce antibodies against α-Gal under the influence of intestinal bacteria, leading to rejection in organ transplants (such as porcine xenotransplantation). This poses a challenge to the activity of exogenous proteins produced in mammalian milk or other body fluids. In contrast, chickens do not produce α1,3-Gal (McKenzie et al., 1999), thus reducing the potential risk of adverse immune responses to drug proteins in eggs produced by genetically modified chickens. A study has analyzed and compared the O-linked glycosylation levels of recombinant human interferon α-2b expressed by transgenic hens. Compared with the naturally produced human interferon α-2b protein, approximately 38% of the target protein was correctly glycosylated (Rapp et al., 2003).

The main component of egg white is protein, accounting for 87% of the total protein mass of the egg. Among them, ovalbumin, ovotransferrin, and ovomucin are the most abundant, with respective percentages of 54%, 12%, and 12%. The relatively low complexity of egg white components is very conducive to purifying the exogenous protein from it. There have been successful cases of processing and purification of certain components of eggs, such as lysozyme (Cao et al., 2015). In 2017, a study employed pseudotyped retroviral vectors to introduce the human erythropoietin (hEPO) gene, under regulatory control, into the germinal disc of newly laid chicken eggs (Stage X), resulting in the production of approximately 90 μg/mL of hEPO in the chicken’s bloodstream (Koo et al., 2017). In recent years, studies have focused on using egg yolk as a bioreactor, expressing recombinant human IgG1 Fc at concentrations ranging from 143.89 ± 55.57 µg/mL to 152.85 ± 85.87 µg/mL (Jin et al., 2023), as well as specific IgY antibodies against the Norovirus VP1 protein (Hadi et al., 2024). These studies collectively demonstrate the significant advantages and developmental potential of chickens as bioreactors. Over the past several decades, numerous studies have demonstrated the utilization of chickens as bioreactors for the expression of foreign proteins in tissues such as serum (Koo et al., 2017), yolk (Felegary et al., 2024), and albumin. The substantial advantages in chicken production in terms of cost, reproduction rates of animal herds, and glycosylation of target proteins have fueled significant advances in the transgenic chicken model as a production system.

For the production of certain proteins that are toxic to mammals, expression in hens represents a favorable alternative. For instance, the expression of human erythropoietin in the mammary glands of rabbits can have harmful effects on them (Massoud et al., 1996), but not in chickens (Steinlein et al., 1994). Eggs serve as an excellent vehicle for recovering therapeutic proteins. Due to the presence of lysozyme in eggs, their contents are sterile, ensuring that proteins remain stable within the egg white. This indicates that therapeutic proteins may have a prolonged half-life when present in egg white (Harvey and Ivarie, 2003). Vaccines for human use have been produced in the eggs of hens for decades, so regulations have been established to help develop regulatory processes for the production of therapeutic proteins in eggs. A significant advantage of using hens as bioreactors compared to utilizing cattle, sheep or goats is the shorter incubation time of 3 weeks and the relatively short generation time of approximately 20 weeks. Consequently, transgenic flocks can be established within a shorter timeframe. A correctly identified transgenic positive rooster can breed approximately 100,000 genetically modified chickens in a year. However, as the company developing this method is still in its nascent stages, there is currently no precise calculation of production costs, although initial estimates suggest they may be competitive with other transgenic animal production systems. In addition, the rapid generation rate and high egg production rate of chicken flocks indicate that the proteins obtained from transgenic hens as a production system could be highly useful for the preparation of pharmaceutical proteins that are currently in great demand in the medical market. For instance, if each transgenic hen produces 100 mg of protein per egg, then this hen can produce about 30 g of medicinal protein per year, which is extremely valuable despite the relatively low feeding costs. Furthermore, the medicinal protein produced by the chicken oviduct bioreactor is secreted in the egg white. This protein is excreted along with the egg from the chicken’s body, ensuring no adverse effects on the animal. Moreover, the gene expression environment in the egg is simple, with the egg white contains a small number of proteins but in high concentrations, and it naturally contains lysozyme. Therefore, this not only extends the half-life of the pharmaceutical proteins expressed in the egg white but also facilitates the subsequent purification of these proteins. Moreover, studies have shown that the medicinal proteins expressed in egg white contain naturally expressed proteins with post-translational modifications, resulting in higher activity and better therapeutic effect (Houdebine L. M., 2002). We have compiled a list of different systems for producing recombinant proteins that are currently under investigation or in use, which is presented as Table 2. The advantages and limitations of these systems have been discussed in the preceding content.

**TABLE 2:** Comparison of different systems for producing recombinant pharmaceutical proteins (Houdebine, 2009).

Points to consider	Production systems							
	Bacteria	Yeast	CHO cells	Transgenic plants	Blood	Milk	Urine	Egg white
production level	++	++	+	++	++	++++	+	++++
Investment cost	+++++	+++++	+	++++	+++	+++	+	+
Production cost	+++++	+++++	++	+++++	++++	++++	+	++
Effect on organism	+++	+++	+++	+++	++	+++	+++	+++
Post-translational modifications	+	++	++++	+++	+++++	++++	+++	++++
Stability of product	+++++	+++++	+++	++++	+++	++++	+++	++++
Scaling up	+++++	+++++	+	++++	++++	++++	+	++++
Purification	+++	+++	++++	+++	++	+++	++	+++
Products on the market	++++	+++	+++++	+	+	++++	+	++

## 3 Current status of avian bioreactor system

In the 1930s, Goodpasture et al. demonstrated that chicken embryos were a suitable vector for producing viral vaccines (Goodpasture et al., 1931). And soon began to be used as a useful tool for a wide range of research in the fields of developmental biology, embryology, toxicity and drug testing, disease modeling, and cell tracking (Stern, 2005; Kain et al., 2014). Since the advent of transgenic technology, chicken embryos have proven to be highly effective tools in the production of transgenic avian species. Subsequent studies have found that transgenic poultry developed in this way can produce recombinant proteins, create disease-resistant varieties, and protect endangered species (Stern, 2005; Kagami, 2016). Therefore, chicken embryo has gradually become an important historical research model in basic and applied science. In 1994, Bj Zeng pioneered the concept of an avian transgenic oviduct bioreactor, which he named the “Gold egg Plan”. He embarked on a study to express exogenous pharmaceutical proteins using the flanking sequences of egg white protein genes. Scholars, research institutions and biotechnology companies around the world have actively engaged in the research of transgenic chicken oviduct bioreactors, prompting continuous innovation in related technologies of transgenic chicken oviduct bioreactors. Researchers have discovered that the particularity of the chicken genome, which is characterized by a high GC content, renders gene editing in chickens more challenging than in other mammals. Additionally, due to the uniqueness of the hen’s reproductive system, there is a notable absence of stable and efficient methods for integrating edited genes into the genome. Hence, the most significant constraint in utilizing transgenic chicken oviduct bioreactors for the production of pharmaceutical proteins lies in the urgent need to develop a technology that is economical, efficient, stable, and easily scalable for the generation of transgenic hens.

To tackle this challenge, early research in transgenic technology primarily targeted cells such as fertilized eggs and blastodisc cells from unincubated fertilized eggs. It also explored methods that directly utilize DNA transduction without relying on pathogen-mediated approaches. Some researchers have constructed vectors containing natural elements conducive to DNA integration, such as transposons and lentiviral vectors, in order to enhance the efficiency of gene integration (Pfeifer, 2006). Lentiviral vectors have been shown to be effective in chickens and mammals (Pfeifer, 2006; Lillico et al., 2007). Research has shown that chimeric genetically modified chickens, generated using non-pluripotent cells, are capable of secreting a monoclonal antibody into their egg whites (Zhu et al., 2005). Recent research has designed a fusion of flexible structures with highly secretory endogenous ovalbumin, significantly enhancing the secretion of exogenous proteins. This approach holds promise for the rapid production of a variety of therapeutic proteins (Xie et al., 2024).

The immense potential of chickens in both industrial production and scientific research has garnered significant attention in the field of avian transgenic research (Han et al., 2015). Despite the technical challenges involved, numerous diverse approaches have been explored to attempt efficient genetic modification of chickens. Several reviews have discussed the different research methods and their relative successes (Ivarie, 2003; Paul and Petitte, 2004; Sang, 2004) Transgenic in poultry makes it possible to produce recombinant proteins, such as therapeutic mAbs (Houdebine, 2009). The modification of avian genomes offers methods and opportunities for studying early embryogenesis, cell tracking, interspecies hybridization, and conserving endangered species (Lee and Han, 2015).

### 3.1 Viral methods

The primary method for obtaining transgenic avian species involves directly injecting viral vectors or DNA donors into the subgerminal cavity (located between the outer blastoderm and the perivitelline layer) of recipient embryos at the X-stage (Salter et al., 1987).The research content related to this process has been described in the previous section. Retroviruses can integrate genes into the chromosomes of host cells, making them prime candidates as vectors for gene transfer. Viral particles are produced by co-expressing the vector RNA genome and genes encoding viral proteins in cultured cells, and then collected from the culture medium. Transgenic avian species can be created using vectors derived from retroviruses. Boerkoel et al. developed an auxiliary cell line and a replication-defective BH-RSV (Bryan strain of Rous sarcoma virus) vector system based on high-titer BH-RSV. Using this combination, they successfully obtained genetically modified chickens with high expression levels of the RSV envelope protein (Boerkoel et al., 1993). At that time, the elements required for specific expression in the oviduct were still unknown. Researchers utilized the ubiquitously expressed promoter, cytomegalovirus (CMV), to express β-lactamase in the serum of genetically modified chickens. A total of three genetically modified chickens were obtained, with β-lactamase concentrations in their serum measured at 5.0 μg/mL, 0.03 μg/mL, and 1.1 μg/mL, respectively. These findings validated the potential of avian species as bioreactor systems (Harvey et al., 2002). Studies have generated genetically modified chickens using an ALV (Avian Leukosis Virus) vector that contains the cytomegalovirus promoter linked to the coding sequence of human interferon α-2b. By purifying and analyzing the eggs from these genetically modified chickens, up to 200 micrograms of human interferon were detected in the egg whites (Rapp et al., 2003). The O-linked glycosylation chains of the recombinant human interferon α-2b expressed in the transgenic hens were analyzed, and it was found that approximately 38% of the recombinant interferon was glycosylated to a degree comparable to that of naturally occurring human protein. Subsequently, a series of gene transfer vectors derived from this have also been developed (Barquinero et al., 2004). High levels of expression of a human single-chain Fv-Fc fusion protein have been achieved in the semen and egg whites of genetically edited chickens using retroviral vectors (Kamihira et al., 2005). Lillico and his team utilized lentiviral vectors constructed based on Equine Infectious Anemia Virus (EIAV), employing the ovalbumin gene’s 5′ regulatory sequence (OVA) as an oviduct-specific promoter. This enabled the production of genetically modified chickens whose oviducts could synthesize functional recombinant therapeutic proteins, namely miR24 and hIFNβ1α. Moreover, the transgenic sequences were successfully integrated into the chickens’ genomes and stably inherited across generations (Lillico et al., 2007). These studies have provided valuable insights into the use of suitable retroviral vector systems for producing transgenic chickens and expressing exogenous proteins. However, despite the many advantages of using retroviral vectors for producing genetically modified chickens, it is difficult to overlook their potential biosafety concerns and some drawbacks in practical production applications. The limited capacity and characteristics of viral vectors restrict the types and sizes of transgenes that can be carried, and they are prone to causing large-scale deletions and chromosomal rearrangements in the genome of recipient animals. Consequently, the germline transmission rate of their transgenic offspring tends to be relatively low. While the CMV promoter is ubiquitous, the expression levels of the reporter gene LacZ driven by the CMV promoter in transgenic hens have been detected to vary considerably among different tissues, with the lowest expression levels observed in the oviduct (McGrew et al., 2004). Furthermore, since viruses lack the ability for targeted modification, it is difficult to achieve precise gene editing using virus-based delivery methods.

### 3.2 Applications of primordial germ cells

In recent years, avian species and related cell lines have played pivotal roles in both basic and applied sciences, the production of recombinant proteins, as well as the development and manufacture of vaccines. PGCs are embryonic cells capable of infinite proliferation *in vitro*. They are incorporated into the bloodstream at the early stages of avian embryonic development (HH14) and then migrate to the embryonic gonads (HH28), where they can be isolated via gonad dissection. This characteristic also provides theoretical feasibility for isolating and transplanting PGCs during early avian embryonic development. The first transgenic bird was produced using PGCs isolated from a chicken embryo at the HH11 stage (L Vick et al., 1993). Since then, the *in vitro* culture and modification of PGCs have undergone significant improvements and advancements in the production of transgenic chickens. PGCs are distinctly identifiable by their apparent size, large spherical nuclei, high glycogen content, and refractive cytoplasmic lipids. They possess robust proliferative capabilities and the ability to differentiate into germ cells *in vitro*. In avian species, the most effective transgenic strategy currently developed is based on genetically modified PGCs. Genetically modified PGCs are injected into the germinal cavity of newly laid fertilized eggs at the X-stage or into the dorsal aorta of recipient embryos at the 14-stage via microinjection. These PGCs then migrate to the gonads of the developing embryos. In Chang et al. (1997) only subjected the collected PGCs to a short-term culture before transplantation. It was until 2006 that scientists successfully achieved long-term culture of PGCs *in vitro*. This research was the first to demonstrate that PGCs could be isolated and cultured *in vitro*, and that they still retained the potential to enter the germline after genetic modification (van de Lavoir et al., 2006). The transgenic avian species obtained through this process become candidates for the production of recombinant proteins. Soon after, the production of recombinant proteins, including therapeutic monoclonal antibodies, using transgenic avian species became feasible (Houdebine, 2009). Initially, the *in vitro* culture of PGCs was quite challenging, and researchers experimented with numerous culture medium formulations. Effective methods have now been developed for culturing chicken PGCs *in vitro* without compromising their germline transmission capability (Song et al., 2014; Whyte et al., 2015; Naito, 2015; Hyung et al., 2016). This advancement has opened up more possibilities for achieving efficient gene modification and precise gene editing. Due to their ability to transmit genetic information to the next-generation, PGCs have gradually emerged as an ideal choice for creating transgenic or gene-edited chickens (Sawicka et al., 2015; Chojnacka-Puchta et al., 2015). The success of genome editing techniques using PGCs in avian species has enabled the development of various gene-edited avian models. These include the production of chickens with specific genes knocked in or knocked out, the preparation of hypoallergenic eggs, and disease-resistant models. Additionally, this technology has made innovative applications such as chicken bioreactors possible.

### 3.3 Gene editing

Traditional transgenic technology often encounters the issue of random integration of foreign genes into the host genome, which may lead to unstable or abnormal gene expression. In contrast, gene editing technology enables site-specific integration of foreign genes, thereby avoiding these problems. These technologies enable researchers to introduce mutations in a precise manner by inducing double-strand breaks (DSBs) (Bibikova et al., 2002; Christian et al., 2010; Jinek et al., 2012; Cong et al., 2013; Cho et al., 2013). In 2013, Schusser et al. reported successful gene editing of PGCs cultured *in vitro* using traditional homologous recombination technology, and obtained the first gene-knockout chicken. They targeted and replaced the J region of the immunoglobulin heavy chain gene with two expression cassettes, β-actin-GFP and CAG-puro, through homologous recombination (Schusser et al., 2013). This research made it possible to produce genetically modified chickens that express humanized antibodies. Subsequently, researchers reported the use of Transcription Activator-Like Effector Nucleases (TALENs) genome editing tools to achieve gene editing in PGCs and obtain individuals with Ovalbumin single-allele modifications. Statistical analysis revealed that approximately 8% of the G1 generation individuals contained mutated Ovalbumin loci, and all G1 generation individuals with Ovalbumin gene mutations were single-allele modified (Park et al., 2014). This result indicated that chicken PGCs could be genetically modified using TALENs technology, and that these modifications could be transmitted through the germline to the next-generation. Although both TALENs and Zinc-finger nuclease (ZFN) rely on the assembly of a series of short protein domains to provide sequence-specific binding to the nuclease domain, and both require paired proteins to complete cleavage at the genomic target site, the TALENs system is, to a certain extent, superior to ZFNs. Compared to traditional homologous recombination methods, novel gene editing strategies have significantly facilitated and enhanced the production of gene-edited animals. The clustered regularly interspaced short palindromic repeats (CRISPR)/Cas9 system only requires the Cas9 nuclease and sgRNA-based nucleotide sequences to ensure the recognition of specific sequences. Changing the target site becomes relatively simple, and multiple sgRNAs can be used simultaneously to target different sites, allowing for gene editing at multiple loci concurrently (Wang Y. et al., 2013). In 2015, a study employed electroporation to introduce plasmids encoding Cas9 and a guide RNA designed to target the transcription factor PAX7 into chicken embryos, successfully demonstrating the efficacy of the CRISPR/Cas9 system in mediating gene editing within avian embryos. This work has paved the way for the application of CRISPR/Cas9 technology in uncovering the molecular underpinnings of bird development (Véron et al., 2015). To date, CRISPR/Cas9 technology has also been successfully applied in chickens. Using CRISPR/Cas9 technology, specific gene editing events can be achieved in chicken somatic cells (Abu-Bonsrah et al., 2016), immortalized fibroblast cell lines such as DF1 cells (Zuo et al., 2016; Bai et al., 2016; Wang et al., 2017), and primordial germ cells (Oishi et al., 2016; Dimitrov et al., 2016). Recently, the CRISPR has been applied to primordial germ cells (PGCs) to disrupt two key egg white genes, OVA and OMUMYID. Oishi and colleagues employed CRISPR in cultured PGCs to generate G1 mutant birds, which were then crossed to produce homozygous oval offspring (G2) (Oishi et al., 2016). Building on these findings, subsequent research has begun to harness the CRISPR/Cas9 system for the targeted knockout of valuable genes in chickens (Zuo et al., 2016; Zhang et al., 2017). Furthermore, Antonova demonstrated that the CRISPR/Cas9-mediated homology-directed repair (HDR) system could be effectively utilized in chicken cell lines to integrate the EGFP gene into the coding sequence under the genomic GAPDH promoter’s control. The efficiency of accurate EGFP gene knock-in, as determined post-drug screening, was remarkably high at 90% (Antonova et al., 2018).The distinctive features of avian embryonic development are intricately connected to advancements in gene-editing techniques, offering substantial promise for the efficient generation of transgenic birds and for establishing a cost-effective and scalable production system for recombinant pharmaceutical proteins (Houdebine, 2009). Recently, researchers have explored the use of immortalized chicken oviduct epithelial (COE) cells as chicken bioreactors for the expression of exogenous genes (Jung et al., 2024; Wu et al., 2024), further demonstrating that the chicken oviduct can be used as an effective tool for the production of exogenous proteins. In recent years, gene editing of avian eggs has been carried out, and different delivery tools have been used to produce different yields of recombinant proteins in eggs with good biological activity (Table 3). The proteins overexpressed in these studies include single-chain fusion proteins, human parathyroid hormone, human erythropoietin, human granulocyte colony-stimulating factor, tumor necrosis factor receptor/Fc fusion protein, green fluorescent protein (GFP), human urokinase-type plasminogen activator, and some antibody proteins. Ovalbumin is a major protein in egg white, which has unique specificity and localized expression characteristics. In 2005, researchers first utilized ovalbumin promoters of 15 kb and 7.5 kb in size to create transgenic chickens. They discovered that the chimeric hens laid eggs that expressed the target protein, with concentrations ranging from 0.5 to 3.4 mg per egg (Zhu et al., 2005). This research outcome demonstrated that the ovalbumin promoter can effectively drive the expression of target proteins in eggs, providing strong support for the development of egg-based bioreactors. Studies have found that the biological activity of human granulocyte colony-stimulating factor (hG-CSF) expressed through chicken oviducts is significantly higher than that of hG-CSF obtained through traditional *E. coli* culture (Kwon et al., 2008). Two other studies found that the biological activity of human erythropoietin was also comparable to that of CHO-derived human erythropoietin (Kodama et al., 2008; Koo et al., 2010). Some researchers have used a 1.9 kb ovalbumin promoter to generate transgenic chickens expressing human erythropoietin (hEPO) in egg white. The concentration of the target protein in the egg white produced by G1 hens is 40.0–55.0 μg/mL, while the expression of hEPO is almost undetectable in their serum. Moreover, the biological activity of the protein collected from eggs is equivalent to that of hEPO obtained by cell culture method (Kwon et al., 2018). These results indicate that specific promoters can achieve expression of target exogenous proteins in a tissue-specific manner, without leakage into the blood and other tissues and organs. They also demonstrate the effectiveness of the ovalbumin promoter and its ability to stabilize the transgenic bioreactor system. With the continuous development and refinement of CRISPR/Cas9 technology, its application prospects in chicken oviduct bioreactors have become even broader. This marks a significant breakthrough in the field of avian bioreactors using gene editing technology. Researchers have successfully utilized this technology to integrate therapeutic protein genes into the chicken genome and achieved stable expression of the target proteins in eggs. These research achievements not only provide strong support for the development of chicken oviduct bioreactors but also offer new ideas and methods for the research and development of other transgenic bioreactors.

**TABLE 3:** Proteins produced in avian eggs (Farzaneh et al., 2017; Rachel et al., 2018).

Promoter	Target Protein	Method	Yield	Year
Cytomegalovirus	β-Lactamase	Avian leukosis virus	0.47–1.34 μg	2002 (Harvey et al., 2002)
Cytomegalovirus	Interferon α-2b (hIFN)	Avian leukosis virus	200 μg	2003 (Rapp et al., 2003)
Ovalbumin	Dansyl hapten mAb	Injection of transformed cES cells	<3 mg	2005 (Zhu et al., 2005)
β-Actin	Single chain Fv-Fc fusion protein	Retroviral vector	5.6 mg	2006 (Kawabe et al., 2006)
Ovalbumin (+ERE)	ScFv-Fc and human interferon B1a	Lentiviral vector	15–50 µg	2007 (Lillico et al., 2007)
Cytomegalovirus	Granulocyte-colony stimulating factor	Moloney murine leukemia virus	<2 mg	2008 (Kwon et al., 2008)
Human PGK	Human erythropoietin	Moloney murine leukemia virus	<1 mg	2010 (Koo et al., 2017)
Ovalbumin	Recombinant Human Interleukin 1 Receptor Antagonist (rhIL1RN)	Lentiviral vector	88.7–233.8 ng	2010 (Kwon et al., 2010)
Ovalbumin	GFP	Recombinant lentivirus	-	2011 (Sung June et al., 2011)
Ovalbumin	Human growth hormone	Retroviral vector	-	2012 (Kodama et al., 2012)
Ovalbumin	Human Neutrophil Defensin 4	Lentiviral vector	1.65–10.18 μg	2015 (Liu et al., 2015)
Ovalbumin (+ERE)	Human lysozyme	Lentiviral vector	57.66 ± 4.10 μg	2015 (Cao et al., 2015)
Ovalbumin	Human erythropoietin	Pseudo typed lentivirus vector	40.1–55.0 μg	2018 (Kwon et al., 2018)
Ovalbumin	hIFN-β	CRISPR/Cas9	∼3.5 mg	2018 (Oishi et al., 2018)
Ovalbumin	GFP	CRISPR/Cas9	-	2020 (Shi et al., 2020)
Ovalbumin	Monoclonal Antibodies	CRISPR/Cas9	1.4–1.9 mg	2021 (Mukae et al., 2021)
Ovalbumin	Human diponectin	CRISPR/Cas9	1.47–4.59 mg	2023 (Kim et al., 2023b)
Ovalbumin	EGFP	CRISPR/Cas9	165.25 ± 19.82 mg	2023 (Kim et al., 2023a)
Ovalbumin	EGFP	CRISPR/Cas9	4.96–9.86 mg	2024 (Xie et al., 2024)
Ovalbumin	Adiponectin (ADPN)	CRISPR/Cas9	0.59 mg	2024 (Yoo et al., 2024a)

Currently, the primary method for preparing gene-edited chickens is through PGCs-mediated technology. However, there are limitations in preparing gene-edited chickens using this method, namely the competition between exogenously transplanted PGCs and endogenous PGCs in the recipient chicken embryo, which decreases the proportion of exogenous PGCs generating gametes and subsequently lowers the preparation efficiency of gene-edited chickens (Ballantyne et al., 2021; Chen et al., 2023). To increase the germline transmission proportion of gene-edited donor PGCs, it is necessary to reduce or eliminate the endogenous PGCs in the recipient chicken embryo, and the development of infertile recipient chicken models can meet this requirement. In recent years, several lines of infertile host tool chickens have been reported. Additionally, since sterile host tool chickens avoid issues such as disease transmission and drug residues in traditional poultry production, their biological products are of higher quality and safety. Furthermore, sterile host tool chickens have a short reproductive cycle, enabling the production of a large number of eggs in a short period of time, which further reduces production costs and simplifies the production process (Marcel et al., 2024).

**FIGURE 2:**
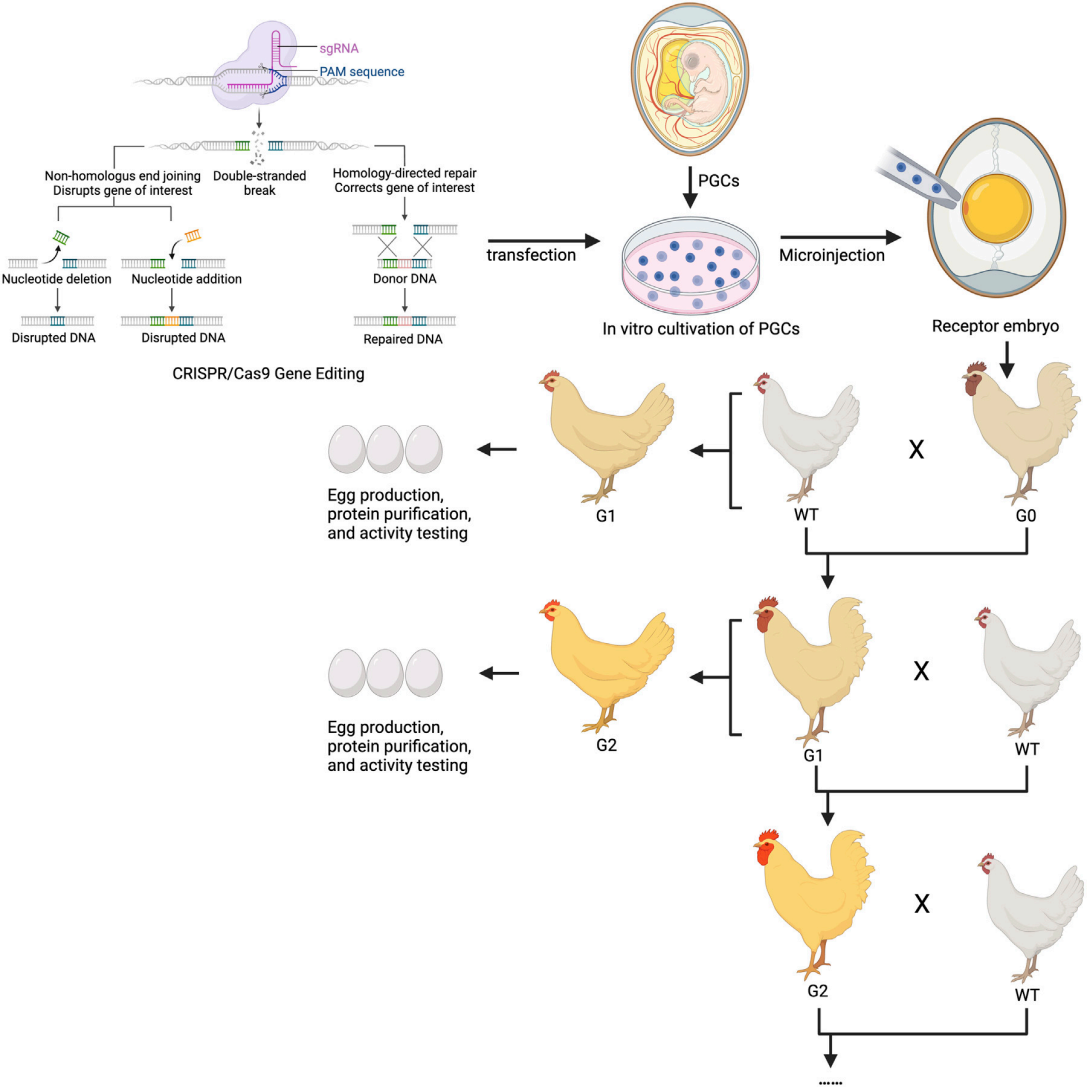
Technical route and breeding strategy for preparing chicken egg bioreactors using gene editing. G1 and G2 generations of gene-edited hens can be used for producing exogenous proteins, while the cocks are used for continuous breeding by passing on the traits. sgRNA, small guide RNA; CRISPR, Clustered regularly interspaced short palindromic Repeats; PGC, Primordial germ cells.

In ongoing research, the methodology and breeding strategy for obtaining gene-edited chickens through PGC editing are largely consistent. After transplanting CRISPR-edited PGCs into recipient chicken embryos to establish the germline chimeric G0 generation, these roosters are used to produce heterozygous mutant genetically modified chickens in the G1 generation, which are then further bred to yield homozygous mutant offspring in the G2 generation. This approach allows for the continuous use of roosters in breeding programs and enables hens to produce exogenous proteins containing the edited genes (Figure 2).

## 4 Discussion and outlook

As the application market for recombinant protein drugs gradually expands, various production methods have emerged in an endless stream, with microbial, plant, and cellular production systems each demonstrating distinct advantages and disadvantages. Considering production costs, time, and protein activity, avian bioreactors offer potential advantages for biotechnology and industrial development (Houdebine, 2009). Significant efforts have been made on germline-modified chickens and cell lines to establish a standard system platform for the production of therapeutic antibodies, recombinant proteins and vaccine production. To date, the feasibility of producing human and veterinary viral vaccines has been achieved in avian pluripotent stem cells and immortalized cells derived from embryos (Farzaneh et al., 2017). Combining cell-mediated transgenic methods, recent advancements in avian stem cells with germline chimeric capability have highlighted the use of avian species as animal models in the pharmaceutical industry. Rapidly advancing gene editing technologies have provided more efficient systems for producing genetically modified chickens, and multiple studies have demonstrated the ability to obtain transgenic avian strains capable of producing biologically active proteins within a relatively short period. Avian bioreactor systems need to be competitive in terms of the time required for protein production, production reliability, cost-effectiveness, and product quality.

The primary application of genetically modified chickens in biopharmaceutical production is the generation of proteins with therapeutic value. These proteins include, but are not limited to, lysosomal acid lipase (LAL), human serum albumin, human high-density lipoprotein, antibodies, and vaccines. The development process typically involves several steps. Firstly, appropriate gene sequences are selected and designed based on the structure and function of the target protein. Secondly, the designed gene sequences are transferred into the nucleus of chicken fertilized eggs using techniques such as microinjection, allowing them to integrate into the chicken genome. Thirdly, genetically modified chickens that stably express the target protein are screened through molecular biological methods. Fourthly, the target protein is extracted from the eggs laid by these genetically modified chickens, and high-purity drugs are obtained through a series of purification steps. Lastly, safety and efficacy assessments are conducted on the extracted drugs to ensure they meet regulatory requirements for pharmaceuticals. Marketing strategies often encompass several key aspects. Firstly, defining the market positioning and target patient population is crucial, based on the function and indication of the target protein. Secondly, promoting brand awareness and reputation is essential, which can be achieved through academic conferences, professional media, social media channels, and other means. Thirdly, establishing partnerships with hospitals, clinics, and other medical institutions helps to broaden sales channels and increase market share. Furthermore, it is crucial to enhance patients' willingness to use the product. This can be achieved by organizing patient education activities and providing them with knowledge on disease prevention and treatment. The production value of transgenic chicken biopharmaceuticals is mainly reflected in several aspects: compared to traditional mammalian cell culture, genetically modified chicken biopharmaceuticals have lower costs. A hen can lay hundreds of eggs per year, and just a few eggs can provide the equivalent of a therapeutic dose of the relevant protein. Additionally, genetically modified chicken biopharmaceuticals provide new drug sources for some rare or difficult-to-treat diseases, helping to meet patients' treatment needs.

Despite significant progress in genetically modified chickens to express exogenous proteins in their egg whites, improvements are still needed. The impact of expressing exogenous proteins on egg integrity in transgenic hens also requires further investigation, which may depend on the nature of the specific protein expressed. In fact, the expression levels of exogenous recombinant proteins in egg whites sometimes are low for unknown reasons, and the eggs produced by gene-edited hens are smaller than wild-type eggs, with relatively lower egg white protein content (Sung June et al., 2011). The site-specific effects have the potential to exert considerable influence on the expression of the target gene. While notable advancements have been achieved in the precise insertion of exogenous genes into the ovalbumin gene locus, targeted editing at this site may inadvertently affect the endogenous expression of ovalbumin. Therefore, the exploration of alternative, safe, and efficient insertion sites for exogenous genes emerges as a pivotal direction for future research endeavors.

In recent years, significant milestone advancements have been achieved in chickens through the utilization of gene editing tools. In practical production, maintaining the stability of transgene transmission across animal generations and ensuring sustainable productivity are equally important. Despite extensive explorations by scientists in the preparation of genetically modified chicken models, many gene editing methods remain challenging in chickens, with low germline transmission efficiency. This is the reason why scientists have been dedicated to developing simple and efficient methods for preparing gene-edited chicken models. The CRISPR system emerges as a simpler, cheaper, and more effective tool, enabling efficient editing of target genes by introducing Cas9 and sgRNA into primordial germ cells (PGCs) through appropriate delivery strategies (Chojnacka-Puchta and Sawicka, 2020). While the CRISPR system boasts numerous advantages, it unfortunately also has some drawbacks. Multiple studies have indicated that chimeric chickens obtained using CRISPR technology and their offspring produce the target protein, but the number of offspring and egg production efficiency are reduced compared to wild-type hens (Oishi et al., 2018). Additionally, the off-target effects of CRISPR cannot be ignored, as they can lead to adverse consequences such as mutations in related sequences in the genome, gene deletions, genomic rearrangements, and oncogene activation, posing potential safety risks to the health of gene-edited animals. Therefore, researchers have conducted studies on the causes of off-target effects in the CRISPR system and proposed measures to reduce off-target efficiency, including designing specific sgRNA sequences and modifying Cas9 proteins (Ran et al., 2013). Furthermore, base editors and prime editors developed on the basis of CRISPR have greatly improved the precision of gene editing and have been preliminarily tested in chicken PGCs (Atsuta et al., 2022). This continuously evolving gene editing technology is expected to further enhance the efficiency of transgenic eggs as bioreactors and reduce off-target effects.

In 2015, the FDA approved the production of human lysosomal acid lipase in eggs (Sheridan, 2016). Existing laws and regulations also facilitate the development of transgenic animals for protein production. However, there remain numerous challenges to address, including achieving optimal protein production levels, ensuring appropriate post-translational modifications of proteins, and purifying them to meet regulatory approval standards. Currently, international legislation concerning transgenic animal needs refinement, and many individuals still harbor concerns about transgenic animals and their derived foods. Therefore, enhancements are required within the legal and institutional realms to refine the oversight and approval processes for transgenic animals and their associated foods and pharmaceuticals. Such advancements will pave the way for a seamless integration of these products into the market, ultimately broadening their application spectrum and enhancing their overall value. In conclusion, genetically modified chickens have the potential to revolutionize the production of protein-based drugs. Their short generation time, high productivity, and ability to undergo appropriate post-translational modifications make them an attractive alternative to traditional production systems. However, before they can be fully utilized as bioreactors, several challenges need to be addressed, including the development of stable and efficient gene editing technologies and the assessment of the impact of exogenous protein expression on egg integrity. With continued research and advancements in biotechnology, it is likely that genetically modified chickens will play an increasingly important role in the production of therapeutic proteins in the future.
